# The Impact of Increased Physical Activity at School on the Nutritional Behavior and BMI of 13-Year-Olds

**DOI:** 10.3390/nu16244329

**Published:** 2024-12-15

**Authors:** Katarzyna Ługowska, Elżbieta Krzęcio-Nieczyporuk, Joanna Trafiałek, Wojciech Kolanowski

**Affiliations:** 1 Faculty of Medical and Health Sciences, University of Siedlce, 08-110 Siedlce, Poland; katarzyna.lugowska.zdoz@uws.edu.pl (K.Ł.); elzbieta.krzecio-nieczyporuk@uws.edu.pl (E.K.-N.); 2 Institute of Human Nutrition Sciences, Warsaw University of Life Sciences, 02-787 Warsaw, Poland; joanna_trafialek@sggw.edu.pl; 3 Faculty of Health Sciences, Medical University of Lublin, 20-400 Lublin, Poland

**Keywords:** adolescents, BMI, obesity, physical activity, diet, nutritional behaviors

## Abstract

**Background/Objectives**: Diet and physical activity (PA) significantly impact health. Unfortunately, a worrying trend of decreasing PA among children and adolescents, accompanied by unhealthy nutritional behavior, is observed worldwide. The aim of the study was to evaluate the nutritional behavior and body mass index (BMI) of adolescents aged 13 years in groups of extended and standard PA at school. **Methods**: The study was conducted in six schools among adolescents born in 2007 (*n* = 213), in two groups of standard and extended PA at school (SPA and EPA), wherein each group received 4 and 10 h of physical education lessons per week, respectively. Their height and body mass were measured. BMI was calculated and compared with percentile charts. A questionnaire was used to assess nutritional behavior and extracurricular physical activity. **Results**: Approximately 62% of adolescents had a normal body weight, with SPA 58%, EPA 64%, overweight 13.63%, SPA 13.75%, EPA 13.50%, obesity 14.13%, SPA 19.25%, EPA 9%, and underweight 11.17%, SPA 8.85%, and EPA 13.50%. Among the potential health-promoting foods, dishes, and beverages, fruits and vegetables were the most frequently consumed items, whereas fish and legume dishes were the least consumed. Butter, white bread, sweets, cheeses, cold cuts, and sausages were all consumed very often. Fast food and lard were the least frequently consumed items. **Conclusions**: A significant proportion of 13-year-olds were overweight or obese. Extended PA had a beneficial effect on BMI, but it had little effect on nutritional behavior among the studied adolescents. The adolescents exhibited moderate nutritional behaviors; however, it was more favorable in those exposed to extended PA at school than those exposed to standard PA. It is possible to argue that extending organized PA at school leads to increased PA during leisure time. It is recommended to increase PA for adolescents by doubling the mandatory number of physical education lessons or other sports activities in the school curriculum.

## 1. Introduction

A healthy diet and regular physical activity (PA) are important elements in improving the health of the young generation [1,2]. Adolescence is a crucial period of physical and mental development, and behaviors that promote regular physical activity and a healthy diet during this period are crucial for health in adulthood [2]. The global prevalence of childhood and adolescent obesity has reached an unprecedented level [3]. The World Obesity Federation estimates that every fourth person in the world will be struggling with obesity by the year 2035 [4]. The steady rise in excess body weight among children and adolescents is particularly concerning. In Poland, the situation is particularly worrying, and studies indicate that Polish teenagers are gaining weight the fastest in Europe [5,6]. One-fourth of teenagers aged 10–19 were found to have excess body weight [7]. Studies estimate that at least 80% of obese school-age children will also develop obesity in adulthood. Physical activity is a crucial component of a healthy lifestyle. The World Health Organization (WHO) recommends regular moderate to vigorous physical activity for an average of 60 min and high-intensity aerobic activity that strengthens muscles and bones at least 3 days per week [8,9]. Studies have shown that regular PA helps develop the musculoskeletal system, cardiovascular and respiratory systems, and nervous system [10]. Increased PA levels in children and adolescents lead to a reduced risk of overweight and obesity and improve body composition, health, and physical fitness [11,12,13,14]. Additionally, studies show that children who engage in regular sports activity exhibit more favorable nutritional behavior compared to children with a more sedentary lifestyle [15,16,17]. Preventing weight gain has focused on factors such as reducing sedentary behavior and energy intake with the diet and expending more energy through regular PA. Despite numerous benefits of PA, adherence rates to PA recommendations among adolescents are poor. A study of 1.6 million people from 146 countries found that over 81% of teenagers do not reach the WHO-recommended level of physical activity [18]. In Poland, only 15% of adolescents aged 11–18 years meet these recommendations [19].

In addition to PA, another important factor influencing health is dietary behavior. There are unique health benefits to the Mediterranean diet, which is recognized worldwide for its healthy eating patterns [20]. It is based on the use of olive oil, eating vegetables, fruits, whole grains, nuts, and seeds, as well as moderate consumption of legumes, fresh fish, and moderate meat consumption. The Mediterranean diet provides most of the necessary macronutrients. In the meantime, young people frequently observe an increase in the consumption of goods high in sugar, fat, and salt, as well as products deep-fried in deep fat, and a decrease in the consumption of goods with high nutritional value [21]. These dietary behaviors are associated with many negative health consequences, such as obesity, hypertension, heart disease, and diabetes [22]. Many countries, including Poland, have dietary recommendations for children and adolescents in graphic form, such as a healthy eating plate, which presents the recommended frequency of consumption of selected food products and beverages [23]. According to the recommendations, the daily diet of school-age children should include vegetables, fruit, wholegrain products, coarse-grained groats and oatmeal, milk and low-fat dairy products, eggs, and other nutrient-dense foods. Lean white meat, legumes, fish, red meat, cold cuts, fatty cheeses (including processed and blue cheeses), wheat bread, fine-grained groats, and fried meat or flour-based meals should be consumed several times a week. Red meat should be replaced with lean white meat, while wheat bread and fine-grained groats should be replaced with products with whole grains and coarse-grained groats [23]. Children and adolescents should drink water as a basic drink. Sweet and salty snacks should be replaced with fresh fruit and vegetables.

Unfortunately, in adolescents’ diets, we can often find undesirable behavior like a low consumption of vegetables, fruits, fish, and wholegrain products, with a high consumption of sweets, salty snacks, and sweet beverages [24]. Hence, the aim of the study was to evaluate the nutritional behavior and body mass index (BMI) of adolescents aged 13 years in groups of extended and standard physical activity at school. The study was intended to show whether children exposed to extended physical activity at school have better nutritional behavior and BMI or not compared to children with standard physical activity.

## 2. Materials and Methods

### 2.1. Participants

This was a pilot, prospective, follow-up cohort study with a control group. The investigation took place between 1 September and 30 September 2021 in six elementary schools in Siedlce, a medium-sized Polish city. The participants were 13-year-old adolescents starting the last grade of elementary school. They all were born in 2007. However, EPA participants began the extended PA program in the fourth grade of school at the age of 10. At the time of this study, the intervention group (EPA) continued their extended PA, i.e., they were exposed to an extended level of PA for a duration of three years compared to the control group. The participants typically went to schools where special classes with extended physical activity lessons had been implemented. Each class had 22–25 children.

The study classified the participants into two groups: the control was those who received four hours of physical education lessons at school. This was a standard dimension of physical education in the school’s curriculum in Poland. The intervention group received ten hours of physical education lessons, which was more than double the standard curricular dimension. We will refer to them as the standard (SPA—standard PA) and extended (EPA—extended PA) groups, respectively. The physical education lessons comprised team games and fitness exercises. The focus was not on coaching specific sports extensively, but on increasing the number of standard physical education lessons in the school curriculum.

We used convenience sampling because we assigned participants to the groups based on their parents’ wishes, and the groups were unequal. The sample size was justified using G*power software, version 3.1.9.7 (University of Kiel, Kiel, Germany) [25].

The inclusion criteria for the study were as follows: (1) good health (no newly diagnosed chronic diseases, injuries, or wounds, and good well-being); (2) age 13 years; (3) consent from the school’s managers, teachers, and parents to participate in the study; (4) ability to complete the questionnaire independently; and (5) not following a special diet that could potentially affect the final result. Failure to meet any of the criteria resulted in being excluded from the study. The research team supervised the completion of the questionnaire about nutritional behavior and anthropometric measurements at the school.

Before the study began, participants received information about the confidentiality of the results and the purpose of the study. The study adolescents verbally agreed to take part, and their parents provided written informed consent. The University of Siedlce Research Ethics Committee approved the research (number 2/2016).

### 2.2. Procedure

The study included an assessment of anthropometric parameters, such as height and weight, as well as participants’ nutritional behavior. A qualified and experienced group of nutritionists carried out the investigation using the same equipment in all schools. The calculated BMI was referred to the percentile charts appropriate for age and gender. The researchers analyzed nutritional behavior based on current guidelines [26,27,28]. Our team conducted anthropometric measurements following the methods outlined in our earlier research [12,16,29,30]. The results presenting comprehensive anthropometric data and nutritional behavior prior to the introduction of extended PA (age of 10 years) were published in our previous paper [12].

An anonymous questionnaire based on the QEB—Questionnaire of Eating Behavior, developed by specialists from the Polish Academy of Sciences, was used to assess the dietary behavior.

The study adhered to the Helsinki Declaration [31]. The researchers asked parents to refrain their children from engaging in any intense physical activity, other than necessary housework, for at least 12 h before the measurements. The measurements took place from 10am to 11am in schools following the necessary guidelines for height and body mass measurements. We used the validated procedures described in our previous work [30].

### 2.3. Body Mass Index

We measured height in centimeters and body mass in kilograms according to the standard procedures [32]. Body height was measured using a Seca 214 stadiometer (Seca GmbH & Co. KG, Hamburg, Germany). Body mass was measured with a Tanita SC-240MA (Tanita Corporation, Tokyo, Japan) [33,34]. BMI was calculated as body weight divided by height (kg/m^2^). We compared the calculated BMI to percentile charts for the population of Polish children [26,27]. According to the criteria, overweight was considered as BMI ≥ 85 percentile, obesity as BMI ≥ 95 percentile, and underweight as BMI ≤ 10 percentile [26,27].

### 2.4. Questionnaire

The survey, conducted anonymously, contained 31 inquiries, each requiring a single response from the aforementioned options. Participants received brief instructions on how to respond to the questions before submitting the survey. Teachers and members of the research team supervised the completion of the questionnaire in classrooms, clarifying any doubts about the questions.

The questionnaire inquired about the frequency of recommendations for healthy diet food products, dishes, and beverages. The first group of questions highlighted foods that should be consumed more often in a healthy diet. These foods included: wholegrain bakery products, buckwheat groats, oatmeal, wholegrain pasta, coarse-grained groats, milk, yoghurts, cottage cheese, white meat dishes, fish, legume dishes, fruit, vegetables, and eggs. These were products of favorable health properties. The second group of questions concerned foods that consumption in a healthy diet should be moderate or low. These foods included: white bread, white rice, white pasta, fine-grained groats, fast food, fried meat or flour dishes, butter, high-fat cheeses, cold cuts, sausages, frankfurters, red meat dishes, sweets, canned meat, sweetened drinks, and energy drinks [28]. Additionally, the survey included questions regarding the regularity of eating breakfast, snacking between meals, and self-assessment of nutrition. The survey also asked about how participants preferred to spend their free time outside of school, including activities like watching TV and using the computer, learning, reading, listening to music, and engaging in physical activities such as sports, walking, and cycling. We used the following categories for consumption frequency and daily frequency (times/day): never; one to three times a month; once a week; several times a week; once a day and several times a day [28].

The results were compared to the principles of healthy eating in force in the country, i.e., the healthy eating plate for children and adolescents [23]. The nutritional behaviors were assessed using a three-point scoring scale: very good, moderate, and poor. The very good score corresponding to healthy nutritional behavior included daily consumption of wholegrain products, thick groats, wholegrain pasta, oatmeal, vegetables, fruit, milk, and fermented products (such as yoghurts), low-fat cottage cheese, and eggs. It also included consumption several times a week of white meats, red meats, fish, legumes, butter, fatty cheeses, wheat bread, wheat pasta, small groats, flour, or fried meat dishes. A very good score was given if sweets, fruit juices, carbonated and energy drinks, fatty cold cuts, sausages, frankfurters, lard, canned meats, and fast food appeared several times a month or not at all. The moderate score corresponding to moderate nutritional behavior included daily consumption of red meat, legumes, butter, fatty cheeses, wheat bread, wheat pasta, small groats, and fried flour or meat meals, as well as consumption several times a week of wholegrain products, thick groats, wholegrain pasta, oatmeal, vegetables, fruit, milk and fermented products, low-fat cottage cheese, fish, and eggs. A moderate score was given if sweets, fruit juices, carbonated and energy drinks, fatty cold cuts, sausages, frankfurters, lard, canned meat, and fast food appeared several times a week. The poor score corresponding to unhealthy nutritional behavior included daily consumption of sweets, fruit juices, carbonated and energy drinks, fatty cold cuts, sausages, frankfurters, lard, canned meats, and fast food. A poor score was given if whole-grain products, large groats, whole-grain pasta, oatmeal, vegetables, fruit, milk, fermented products, low-fat cottage cheese, white meats, or fish and eggs appeared in the diet once or several times a week, and red meats, butter, fatty cheeses, wheat bread, wheat pasta, small groats, fried flour, or meat meals appeared in the diet once or several times a day.

### 2.5. Statistical Analysis

Statistical calculations were performed using Microsoft Excel 365 (Microsoft, Corp, Washington, DC, USA) and Statistica 13 (Stat Soft, Krakow, Poland). The significance level was alpha ≤ 0.05. We conducted various statistical tests: the *t*-test, the Mann–Whitney U test, and analysis of variance. We conducted this analysis after checking for the normality of the distribution and the homogeneity of variance. The Shapiro–Wilk test was used to check the normality of the distribution, and the Levene test was used to verify the assumption of homogeneity of variance for values greater than the accepted significance coefficient of 0.05. To analyze the interdependence of BMI and PA at school and nutritional behavior, we performed a PCA analysis (principal component analysis).

## 3. Results

### 3.1. Group Characteristics

The study obtained consent from 236 parents and adolescents, consisting of 111 EPA and 125 SPA. However, the study included 213 participants, 99 EPA (girls 50; boys 49) and 114 SPA (girls 56; boys 58). The reason for the exclusion was the absence of the participant on the day of measurement. The study excluded approximately 11% of EPA participants and 9% of SPA participants. The average age of the participants was 13.69 years, and they were similar in terms of socioeconomic status and backgrounds.

### 3.2. Anthropometric Measures

The body mass, height, BMI of participants, along with the corresponding percentiles, after 3 years of increased PA are shown in Table 1. Participants were similar in height (SPA 167.23 cm; EPA 166.86 cm; *p* = 0.148). Boys were taller on average than girls (169.76 cm vs. 164.67 cm, respectively; *p* = 0.021). Mean body mass was higher in SPA than EPA (61.24 kg vs. 58.39, respectively; *p* = 0.002). Girls were lower than boys (57.19 kg vs. 62.58 kg, respectively; *p* = 0.031). EPA adolescents had a lower BMI on average than SPA (20.84 kg/m^2^ and 75th percentile vs. 21.70 kg/m^2^ and 50th percentile, respectively; *p* = 0.012). On average, BMI was slightly higher in boys than in girls (21.53 kg/m^2^ vs. 21.06 kg/m^2^, respectively; *p* = 0.147).

At the beginning of the study (age of 10), the mean height for the entire group was 142.96 cm (SPA 143.37 cm; EPA 142.55 cm; *p* = 0.350). After 3 years, the height increased by 24.28 cm in EPA and 23.13 cm in SPA (*p* = 0.120) [12]. The height of girls in EPA increased by 21.52 cm, while that of boys increased by 27.11 cm (*p* = 0.021), and in SPA, the height increased by 22.49 cm in girls and 25.97 cm in boys (*p* = 0.025) [12].

Over the 3-year period, the average weight gain increased from 37.32 kg to 58.36 kg EPA (an increase of 21.04 kg), while in SPA from 38.31 kg to 61.24 kg (an increase of 22.93 kg) [12]. The average weight gain over the 3-year period was almost 22 kg (*p* = 0.010). In EPA girls, the weight increased from 37.79 kg to 55.60 kg, while in SPA it increased from 37.10 to 58.61 kg. In the EPA boys, the weight increased from 36.86 kg to 61.17 kg, in SPA from 39.35 kg to 63.78 kg.

Before extended PA was introduced, the BMI was 18.26 in EPA and 18.41 in SPA. The BMI of EPA girls increased from 18.37 to 20.48 (*p* = 0.010), while that of boys from 18.15 to 21.20 (*p* = 0.041). The BMI of SPA girls increased from 18.20 to 21.58 (*p* = 0.001), and of boys from 18.60 to 21.81 (0.010) [12].

### 3.3. BMI Categories

**Figure 1:**
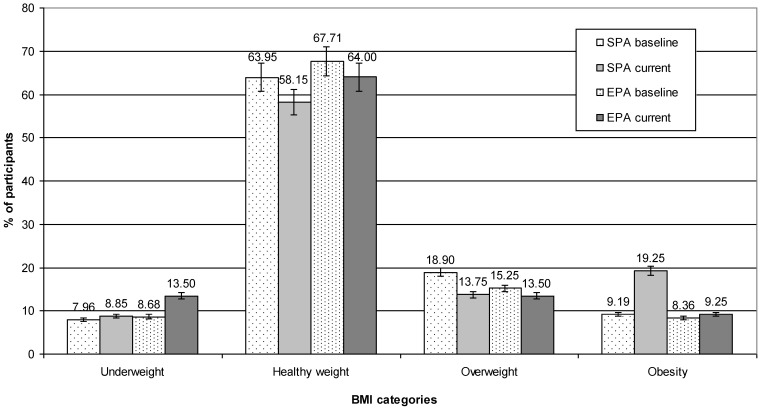
BMI categories of studied adolescents at baseline (the year 2017) and currently (2021). EPA—elevated PA group; SPA—standard PA group.

Almost 62% of participants had a normal body weight (SPA 58% vs. EPA 64%; *p* = 0.001), overweight in 13% (SPA 13%, EPA 13%; *p* = 0.067), obesity in 14% (SPA 19% vs. EPA 9%; *p* = 0.000) and underweight in 11% (SPA 9% vs. EPA 13%; *p* = 0.000) (Figure 1). Excess body weight (overweight and obesity) was more common in SPA than EPA (33% vs. 22%, respectively; *p* = 0.001).

Before the introduction of extended PA at school (in the year 2017), 64.75% of EPA children were characterized by normal body weight, 18.86% were overweight, 7.37% were obese, and 10.66% were underweight [12]. After three years of extended PA (the year 2021), the number of EPA participants with excessive body weight (overweight and obesity) decreased from 26.23% to 22.50% (*p* = 0.021) [12]. At the age of 10 years, 66.55% of the participants were of normal body weight, 13.31% were overweight, 10.45% were obese, and 9.69% were underweight. After three years of standard PA at school, the number of SPA participants with excessive body weight (overweight and obesity) increased from 23.76% to 33% (*p* = 0.010) [12].

Obesity was most frequently noted in SPA girls (21%) and was twice as high as in EPA girls (10%). Whereas in boys, obesity was twice as frequent in SPA than EPA (17% vs. 8.00%, respectively; *p* = 0.001). The overweight rate was similar in all groups but most frequently occurred in SPA boys and EPA girls (15% and 12%, respectively; *p* = 0.002). Underweight was most frequent among EPA boys (15%) and was twice as frequent as in SPA boys (7%), but in girls it was similar in both groups (12% vs. 10%, respectively; *p* = 0.050) (Table 2).

After three years of extended PA at school intervention, with a break for lockdown during the COVID-19 pandemic (the year 2021), in the EPA girls, a decrease in excessive body weight (overweight and obesity) from 27.87% to 25% and an increase in normal body weight from 60.66% to 63% were observed. In relation to the baseline (in the year 2017), in the EPA boys, a decrease in excessive body weight (overweight and obesity) from 21.31% to 20% was observed, and a decrease in normal body weight from 68.85% to 65% was observed. Consequently, an increase in underweight from 9.84% to 15% was noted. In the SPA girls, an increase in excessive body weight from 26.31% to 34% and a decrease in normal body weight from 64.91% to 55.30% were observed [16]. Among the SPA boys, a decrease in normal body weight from 68.19% to 61% and an increase in excess body weight from 21.21% to 32% were observed [12].

### 3.4. Nutritional Behavior

Table 3 and Table 4 show the declared frequency of food consumption (in the year 2021), after three years of PA intervention in the EPA group. Adolescents most often ate 4 meals a day (EPA 40%, SPA 32%; *p* = 0.001), and only 21% of children ate 5 meals (SPA 24%, EPA 17%; *p* = 0.001). Boys more often ate 5 meals compared to girls (23% vs. 19%, respectively; *p* = 0.024). Nearly 62% of participants ate breakfast every day (SPA 58%; EPA 66%; *p* = 0.470). However, 42% of SPA and 34% of EPA ate breakfast irregularly. Girls more often declared regular breakfast consumption than boys (65% vs. 59%, respectively; *p* = 0.012).

On average, participants were characterized by moderate nutritional behavior (SPA 52%; EPA 56%; *p* = 0.081). A very good score was given to 24% of EPA and 20% SPA participants (0.074). A poor score was given to 20% EPA and 28% SPA participants (0.066). EPA participants showed, on average, very good nutritional behavior in terms of declared daily consumption of milk (42%), vegetables (61%), and fruit (69%). Approximately 73% of children did not drink energy drinks at all, 78% did not consume canned meat, and 95% lard. 24% of children scored very well. Moderate nutritional behavior included consumption several times a week of white meat (71%), red meat (58%), fried meat and flour meals (78%), fatty cheeses (62%), wholegrain bread (41%), coarse groats (74%), fine groats (74%), cold cuts (49%), cottage cheese (50%), fermented milk products (66%), and eggs (69%). Fish was consumed most often once a week (56%). On average, 41% of EPA participants declared consumption of carbonated drinks 1–3 times a month, and 22% not at all. Daily consumption of wheat bread was 74%. A moderate result was obtained by 56% of EPA children. Poor nutritional behaviors of EPA participants concerned the consumption of sweets several times a week (38%), fruit juices (63%), and fast food products (50%). Poor nutritional behavior (consumption 1–3 times a month) was noted in the consumption of legumes (39%). Daily consumption of butter was declared by 54% of participants. Unhealthy nutritional behavior was noted in 20% of EPA participants. The moderate scores were obtained by 52% of EPA participants.

SPA participants showed very good nutritional behaviors in declared daily consumption: vegetables (56%), fruit (59%). 60% did not consume canned meat, and 89% lard. 65% of children did not drink energy drinks at all. A very good score was obtained by 20% of SPA participants. The moderate score included the declared consumption of several times a week by SPA participants: white meat (79%), red meat (74%), fried meat and flour dishes (68%), yellow cheese (66%), wholegrain bread (49%), coarse groats (69%), fine groats (81%), cottage cheese (50%), fermented milk products (58%), milk (61%), and eggs (76%). Fish was consumed most often once a week (49%). Daily consumption of wheat bread was 81%. Poor nutritional behaviors in SPA participants concerned the consumption of sweets (54%) and cold cuts (39%) several times a day. Poor nutritional behavior included the consumption of fruit juices (66%) and fast food products (66%) several times a week. Low consumption of legumes (44%) was noted. Approximately 59% of participants consumed butter every day. On average, 21% of participants consumed carbonated drinks several times a week. Unhealthy nutritional behavior was noted among 28% of participants.

Approximately 24% of girls demonstrated very good nutritional behavior. It concerned daily consumption of milk (64%), vegetables (74%), and fruit (77%). Approximately 76% of girls did not drink energy drinks at all, 70% did not consume canned meats, and 90% did not consume lard. The girls’ good nutritional behavior included eating several times a week: white meat (73%), red meat (66%), fried meat and flour dishes (72%), fatty cheeses (64%), wholegrain bread (41%), coarse-grained groats (70%), fine-grained groats (74%), cold cuts (48%), cottage cheese (49%), fermented milk products (62%), and eggs (69%). Fish was eaten most often once a week (42%). On average, 39% of girls declared consumption of carbonated drinks several times a week. Daily consumption of wheat bread was declared by 73% of girls. Moderate results were obtained by 56% of girls. The girls’ poor nutritional behavior concerned eating sweets several times a day (44%), fruit juices (62%), and fast food products (55%). Poor nutritional behavior (consumption 1–3 times a month) was noted in the declared consumption of legumes (40%). Daily consumption of butter was declared by 53% of girls. Unhealthy nutritional behavior was observed in 20% of girls. Approximately 20% of boys demonstrated very good nutritional behavior (vs. 24% of girls, *p* = 0.069). It concerned daily consumption of vegetables (55%) and fruits (58%). Approximately 68% of boys did not consume canned meats, and 94% lard. 62% of boys did not drink energy drinks at all.

Moderate nutritional behavior was noted in 52% of boys (vs. 56% of girls, *p* = 0.085), and very good 20%, respectively (vs. 24%, *p* = 0.069). Moderate scores included consumption several times a week of white meat (76%), red meat (71%), fried meat and flour meals (73%), fatty cheeses (63%), wholegrain bread (49%), coarse-grained groats (71%), fine-grained groats (80%), cottage cheese (51%); fermented milk products (62%), milk (62%), and eggs (75%). Fish was consumed most often, once a week (52%). Daily consumption of wheat bread was 82%. Moderate results were obtained by 52% of boys. Poor dietary behaviors concerned the consumption of sweets (39%) and cold cuts (50%) several times a day. Poor behaviors included the consumption of fruit juices (61%) and fast food (62%) several times a week. Low consumption of legumes (43%) was noted. 60% of children consumed butter daily. On average, 44% of boys declared drinking carbonated drinks several times a week. Unhealthy nutritional behavior was noted in 28% of boys (vs. 20% of girls, *p* = 0.092).

Snacks between main meals were eaten by approximately 82% of participants (18% SPA and 18% EPA; *p* = 0.170). Boys consumed snacks slightly more often than girls (81% vs. 83%, respectively; *p* = 0.062). Participants usually ate salty snacks (SPA 31%; EPA 27%; *p* = 0.071), fruits (SPA 22%; EPA 29%; *p* = 0.010), and sweets (SPA 23%; EPA 23%; *p* = 0.715). Girls consumed sweets more often than boys (24% vs. 21%, respectively; *p* = 0.871) and salty snacks (32% vs. 26%, respectively; *p* = 0.610). The most popular drink was water (SPA 76%; EPA 65%; *p* = 0.004). Tea without sugar was drunk by 10% EPA and 4% SPA (*p* = 0.001).

On average, 37% of participants reported consuming dairy products once a week (SPA 38%; EPA 36%; *p* = 0.415). Boys consumed milk more often than girls (Table 4). Yoghurts (SPA 41% and EPA 46% (*p* = 0.221), cottage cheese (SPA 21% and EPA 30%; *p* = 0.000), and cheese (SPA 43%; EPA 41%; *p* = 0.078) were also consumed once a week (Table 3). Boys more often consumed yoghurts than girls (46% vs. 41%, respectively; *p* = 0.061), cottage cheese (29% vs. 22%; *p* = 0.078), and cheese (44% vs. 40%; *p* = 0.072).

The study found that a high percentage of adolescents, averaging 77%, chose to eat white bread at least once a day. Significantly more SPA than EPA chose white bread (81% vs. 74%, respectively; *p* = 0.431). Boys consumed wheat bread more often than girls (82% vs. 73%; *p* = 0.010). Participants consumed wholegrain cereal products rarely, with only 21% of participants consuming them every day (SPA 19%; EPA 23%; *p* = 0.001). The consumption of wholegrain bread once a day was declared by 24% of girls and 18% of boys (*p* = 0.023).

Only 6% of participants consumed fine-grained groats, oatmeal, and whole-grain pasta at least once a day (SPA 4% and EPA 9%; *p* = 0.012). Several times a week, these products were eaten by 17% of girls and 25% of boys (*p* = 0.028). More than ^2^/_3_ of participants consumed white rice, fine-grained groats, and white pasta (SPA 61% and EPA 55%; *p* = 0.611). The frequency of these products consumption in girls and boys was at a similar level (*p* = 0.920).

The frequency of poultry consumption was moderate, but on average, EPA participants reported it more often than SPA. Almost 38% of participants consumed poultry several times a week (SPA 36%, EPA 40%; *p* = 0.024). Boys showed a higher consumption of poultry compared to girls (37% vs. 30%, respectively; *p* = 0.035). Adolescents ate poultry more often than red meat, with the latter consumed most often once a week, on average 49% (SPA 56%, EPA 45%; *p* = 0.041). Consumption of red meat several times a week was declared by 12% of girls and 20% of boys (*p* = 0.020). Participants reported a high consumption of cold cuts, sausages, and frankfurters. Approximately 39% of SPA and 35% of EPA participants ate the above products at least once a day (*p* = 0.047). Boys significantly more often declared consumption of these foods than girls (50% vs. 24%, respectively; *p* = 0.001). Whereas the frequency of fish consumption was low, with rates of once a week being 52% (SPA 49% and EPA 56%; *p* = 0.021) and once a month 32%, while 14% did not eat fish at all. Fish consumption was low regardless of the group type; however, EPA participants ate significantly more fish than SPA. As many as 20% of girls and 13% of boys did not eat fish at all (*p* = 0.047).

Fruit and vegetables were more frequently eaten by EPA participants than SPA. Every day, vegetables were consumed by 56% SPA and 61% EPA participants (*p* = 0.027). Girls consumed vegetables more often than boys (62% vs. 55%; *p* = 0.023). Fruit was consumed every day by 59% SPA and 69% EPA (*p* = 0.003). Girls consumed fruit more often than boys (69% vs. 58%, respectively; *p* = 0.010).

Butter was the most frequently chosen fat, with 59% of SPA participants and 54% of EPA participants eating it every day (one or several times) (*p* = 0.044). Approximately 53% of girls and 60% of boys declared every day butter consumption (*p* = 0.028), and 22% of girls and 17% of boys several times a week.

The most frequently declared fast food consumption was once a week. This indicated 50% of participants (SPA 53%; EPA 47%; *p* = 0.041), while 1–3 times a month was indicated by 33% SPA and 45% EPA. Boys consumed fast food more often than girls (*p* = 0.043).

The study observed a high consumption of sweets. Approximately 54% SPA and 29% EPA participants ate sweets every day (*p* = 0.001), and almost 30% ate sweets several times a week (SPA 22%; EPA 36%; *p* = 0.020). Consumption of sweets was at a similar level in girls and boys (*p* = 0.240).

Participants typically chose juices once a week (SPA 45%; EPA 47%; *p* = 0.065), more often in boys than girls (21% vs. 17%, respectively; *p* = 0.61). Sweetened carbonated drinks were drunk several times a week by 19% SPA and 9% EPA participants (*p* = 0.003). Participants drank energy drinks most frequently, 1–3 times a month. That was reported by 24% SPA and 19% EPA participants (*p* = 0.062). However, 65% SPA and 73% EPA did not drink energy drinks at all. Boys significantly more often drank carbonated drinks than girls (*p* = 0.020) and energy drinks (*p* = 0.038).

Compared to the initial measurements (baseline at the age of 10), changes in nutritional behavior were noted in both EPA and SPA. In SPA, the declared consumption of milk and fish was at a similar level compared to the measurements at the age of 10. An increase in the consumption of fruit was noted (from 42% to 59%), as well as vegetables (from 39% to 56%). The consumption of wheat bread was still higher compared to wholemeal bread, with an increase in the consumption of wholemeal bread from 7% to 19%. The consumption of fine-grained groats once a week increased from 46% to 61%, while the consumption of coarse-grained groats decreased from 57% to 43%. In SPA, an increase in the declared consumption of cold cuts and sausages was observed from 30% to 39%. White meats were still more often chosen than red meats. Fast food was definitely more popular among 13-year-olds, with an increase from 35% to 53%, and the frequency of eating sweets also increased from 31% to nearly 50% [16].

EPA participants showed an increase in the declared consumption of milk (from 31% to 42%) and fruit (from 33% to 69%). Vegetable consumption increased significantly (from 52% to 61%). Whole-meal bread consumption increased from 13% to 23%, and wheat bread consumption from 71% to 74%. There was a decrease in the consumption of coarse-grained groats and oat flakes (from 88% to 74%), fine-groated groats and white meal pasta (from 79% to 74%), as well as an increase in the consumption of white meats (from 46% to 71%) and red meats (from 49% to 64%). Fish consumption was still very low. Over the three-year period, the participants increased consumption of fast food (from 41% to 47%) and sweets (from 29% to 57%) [16].

### 3.5. Extra-Curricular PA

The surveyed adolescents most often declared that they spent their free time practicing in various forms of PA. On average, 69% declared active forms of spending free time (SPA 64%, EPA 75%; *p* = 0.003), while 31% declared sedentary activities, such as watching TV or Internet surfing (SPA 36%, EPA 25%; *p* = 0.027). Boys, significantly more often than girls, declared active forms of spending free time (79% vs. 60%, respectively; *p* = 0.001).

**Figure 2:**
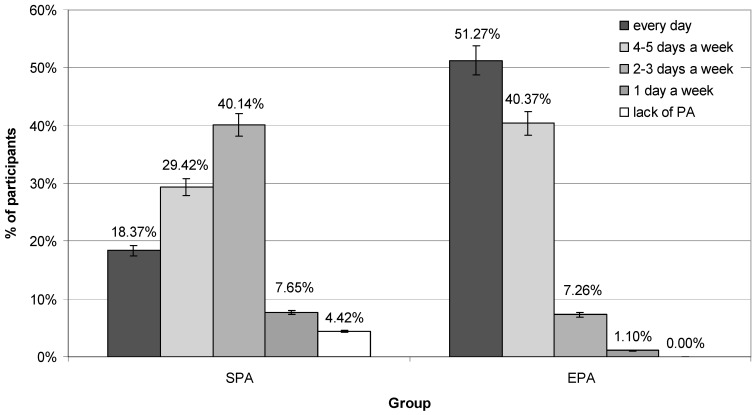
Declared extra-curricular physical activity (the year 2021). EPA—extended PA group; SPA—standard PA group.

EPA adolescents were more likely to participate in extra-curricular PA than SPA (Figure 2). Approximately 91% of EPA participants undertake extra-curricular PA at least 4 days a week, while in SPA it was almost 47%. Approximately 35% of them reported every day extra-curricular PA during their free time (EPA 52% vs. SPA 18%, respectively; *p* = 0.000). EPA participants were more likely to report being physically active four to five days a week (EPA 41% vs. SPA 29%; *p* = 0.000). While SPA participants were more likely to be physically active two or three days a week (SPA 40% vs. EPA 7%; *p* = 0.000). On average, 4% of SPA adolescents reported taking part in extra-curricular PA only one day a week. Approximately 1% of EPA and 12% of SPA adolescents reported only one day a week or a lack of extra-curricular PA. Approximately 42% of boys and 28% of girls reported being physically active every day (*p* = 0.010), while 35% reported being physically active 4–6 days a week (33% and 37%, respectively; *p* = 0.047).

The vast majority (EPA 94% and SPA 97%; *p* = 0.817) were fully aware of the beneficial effects of a healthy diet on sports performance. Most adolescents declared their diet to be healthy, with SPA 60% and EPA 59%, while 40% considered it to be unhealthy.

### 3.6. PCA

In PCA, we compared variables as nutritional behavior (variables A and B), BMI (variable C), and the level of PA at school (variable D). We split the nutritional behavior into two indicators. The first indicator (variable A) reflected the average consumption of foods such as vegetables, fruits, wholegrain products, coarse-grained groats, cottage cheese, yogurts, milk, lean meat, eggs, and water, all of which are recommended in a healthy diet. The second (variable B) concerned average consumption of those foods whose intake should be moderate or low (white bread, fine-grained groats and wheat pasta, rice, fried meat or flour dishes, red meat, sausages, canned meat, fatty cheese, sweets, fast food products, and carbonated drinks and juices).

**Figure 3:**
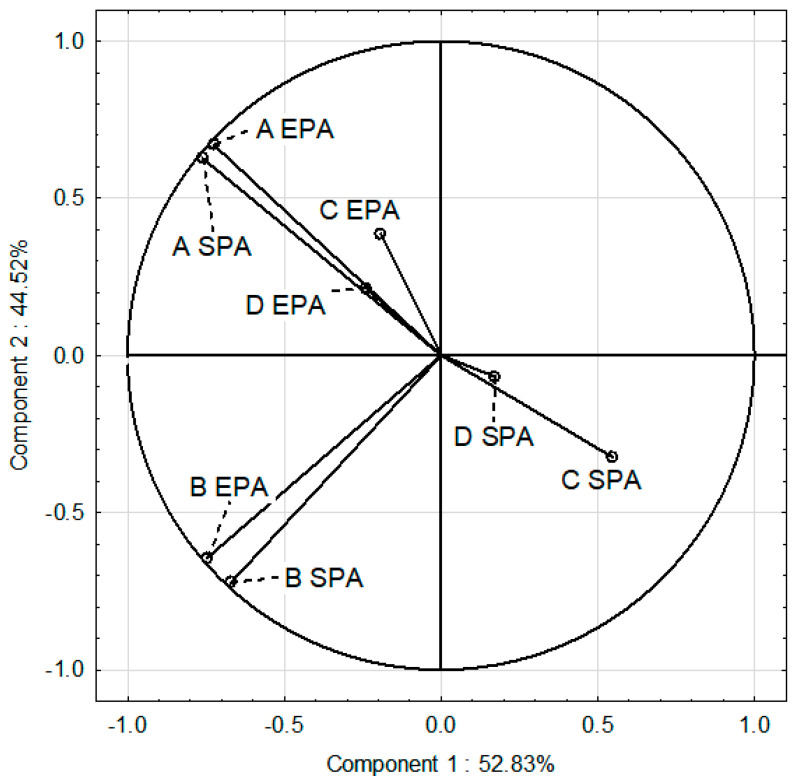
PCA analysis of physical activity (PA) at school, BMI and nutritional behavior correlations in SPA and EPA groups. EPA—extended PA group; SPA—standard PA group; A EPA—favorable nutritional behavior in EPA; B EPA—unfavorable nutritional behavior in EPA; A SPA—favorable nutritional behavior in SPA; B SPA—unfavorable nutritional behavior in SPA; C EPA—normal BMI in EPA; C SPA—normal BMI in SPA; D SPA—PA in SPA; D EPA—PA in EPA.

In the SPA group, BMI (variable C) and favorable nutritional behavior (variable A) were one group of variables positively correlated with each other. Unfavorable nutritional behavior (variable B) constituted another group of variables. Standard PA at school was associated with an increase in unfavorable nutritional behavior (B) and BMI (C) (Figure 3). SPA adolescents consumed fewer health-promoting foods (A) and more foods that should be limited in a healthy diet (B) than EPA adolescents. Whereas, in the EPA group, PA at school (D), BMI (C), and favorable nutritional behavior (A) constituted one group of variables positively correlated with each other. Similarly to SPA, unfavorable nutritional behaviors (B) constituted another group of variables. Expanded PA at school positively correlated with normal BMI and more favorable nutritional behavior and reduced unfavorable nutritional behavior (Figure 3).

The results of the PCA revealed a negative correlation between standard PA at school and favorable nutritional behavior and a healthy BMI. Conversely, extended PA at school was positively correlated with normal BMI and more favorable nutritional behavior among adolescents. It also negatively correlated with unfavorable nutritional behaviors.

## 4. Discussion

The study showed that extended PA at school reduced the number of adolescents who were obese and improved their nutritional behavior slightly. This is in agreement with previous studies, which also found that low physical activity is often positively associated with overweight and improper nutritional behavior [12,13,14].

Our observations suggest that children and adolescents who are physically active tend to have a lower BMI than children who have low PA. This is consistent with our observations. We found that most teenagers were healthy and had a higher percentage of EPA than SPA (64% vs. 58%, respectively). Nowaczyk et al. (2023) showed that after 10 months of increased PA, the percentage of participants with a normal BMI increased from 52% to 58%, while in the group without additional PA it decreased from 59% to 49% [35]. In our study, we found that the prevalence of excess body weight in 13-year-olds was very high, with an average of 28% being overweight or obese, including more often in SPA than EPA. Data from a few years back show that about 22% of teenagers are overweight [24]. Our research indicates that the percentage is increasing. The study by Nowaczyk et al. (2023) also shows that almost 28% of 11-year-olds are overweight [35]. Nonetheless, lower levels of overweight and obesity were noted in the studies conducted by Zou et al. (2023) [36] and Bartosiewicz et al. (2020) [37]. Similar findings were reported by Petri et al. (2020) and Lima et al. (2021) [38,39]. According to the cited article, 22% of children performing sports were overweight or obese [38].

According to Jacob et al. (2021), interventions that focus on regular PA and healthy eating have the greatest potential to reduce BMI in adolescents to normal values [40]. This is in line with our results. This finding is also consistent with the study by Gbska et al. (2023), which showed that higher BMI values were observed in the group of adolescents not participating in additional PA [41]. Nowaczyk et al. (2023) demonstrated that following a period of 10 months of increased physical activity, the percentage of overweight participants decreased from 25% to 17%, and with obesity, the percentage decreased from almost 3% to less than 1% [35]. Despite their increased physical activity at school, the teenagers in the study still had a high share of overweight and obesity. There were 22% of extended PA participants who were overweight and obese compared to 33% of those who had standard PA. Nonetheless, several studies have failed to demonstrate the impact of elevated PA on body mass index [42,43].

In recent years, it has been frequently reported that in many nations, the likelihood of obesity is higher among teenage boys compared to girls [44]. In our study, we found that excess body weight was more often found among girls than boys (average 29% vs. 26%, respectively). The same result was shown in the study by Bartosiewicz et al. (2020), (21% vs. 23%, respectively) [37]. The study by Nowaczyk et al. (2023) revealed that excess body weight was more prevalent in females than males [35]. Unfavorable nutritional behavior and low PA are associated with the risk of excess body weight. Schlesinger et al. (2019) found that certain food groups are associated with the risk of excess body weight, such as sweets, salty snacks, and highly processed foods [45]. A healthy diet for adolescents should include vegetables, fruit, legumes, whole grains, milk, fish, eggs, and lean meat [20,21,46].

Our results showed that adolescents who were exposed to extended PA at school had more favorable nutritional behavior than those who were exposed to standard PA. Several significant studies have also demonstrated correlations between sports and generally healthier dietary choices, including a reduction in consumption of sweets, sugar-sweetened beverages, and energy drinks, akin to our findings [17,47,48]. A small group of teenagers adhered to the daily allowance of whole grains and vegetables. An even smaller percentage of the study participants consumed fish, legumes, coarse-grained groats, wholegrain pasta, and oatmeal, which could have contributed to the higher BMI. The dietary irregularities and qualitative nutritional mistakes found in this study were, in fact, similar to those reported in other studies [38,39].

Our study found that girls were more likely to consume milk, wholegrain bread, coarse-grained groats, oatmeal, lean meat, fruit, and vegetables than boys. This was consistent with national observations, which showed that girls had greater nutritional knowledge than boys [49]. On average, vegetables and fruits were consumed every day, with fruits being more popular than vegetables. It is worth mentioning that adolescents who were exposed to extended PA at school more often consumed vegetables and fruit compared to those of standard PA. These results are similar to the findings of Kanellopoulou et al. (2021), who found that participation in additional sports activities may be associated with an increased consumption of fruits and vegetables [50]. Contrary to our investigation, Froberg et al. (2002) demonstrated that participation in organized PA during free time was not associated with a greater intake of fruit and vegetables [51].

In our study, the declared frequency of consumption of milk, yogurt, and cottage cheese was low, with EPA adolescents consuming them more often than SPA. This is consistent with Fröberg et al.’s (2022) observations [51]. We also observed a high consumption of wheat bread and a low consumption of wholegrain products and coarse-grained groats, such as oatmeal. EPA participants ate wholegrain products more often than they did SPA, but their consumption was still low. Hurr et al. (2012) also found that children and adolescents do not eat enough wholegrain products [52]. Frequent consumption of sweets and fast foods is one of the most detrimental nutritional habits among children and adolescents. We observed a high consumption of the above products in our study. Sweets were eaten daily by 54% of SPA participants and 29% of the EPA participants. Fast food, on the other hand, was favored by 53% of SPA and 47% of EPA once per week. Archero et al. (2018) found that teenagers every day ate sweets or frequently went to fast-food restaurants [53]. The high consumption of fast food was noted in the work of Scaglioni et al. (2018), where 10% of teenagers declared daily consumption and 41% once a week [54]. It was noted that a high consumption of sweet juices and carbonated drinks was noted, with nearly 30% of participants drinking juices several times a week and 46% drinking once a week. Carbonated drinks were drunk by 14% and 28%, respectively. It is noteworthy that juices and carbonated beverages were consumed more frequently in SPA than in EPA. Vega-Ramrez (1924) demonstrated a higher consumption of carbonated drinks [55].

We found that EPA adolescents were much more likely than SPA adolescents to engage in additional, out-of-school PA, with boys more often than girls. Similar results were reported by Chen et al. (2018), who found that adolescents who were more active at school and in extracurricular activities had significantly higher levels of moderate PA during the week. This was confirmed in our work [56]. It is possible to argue that increasing organized PA at school leads to even more PA during leisure time. Other studies also observed that boys engage in more physical activity than girls [57,58]. The study found that SPA adolescents were more likely to engage in sedentary activities than EPA adolescents. The study by Warnberg et al. (2021) showed that more time spent in front of the screen was associated with lower consumption of healthy food, including vegetables, legumes, fish, and nuts, and higher consumption of sweets and fast food [59].

The present study found that the amount of organized PA at school was a factor related to BMI and nutritional behavior of participants. A systematic review by Lehmann et al. (2020) indicated that regular PA can help prevent obesity [60]. Other studies showed a positive association between PA and favorable nutritional behavior [61]. However, some researchers did not confirm these relationships [62].

Our study has some limitations. First, the study was restricted to a relatively small group of participants. The investigation should be extended to adolescents from other ages and from a broader geographical area. The content of body fat, which appears to be more suitable for determining the degree of overweightness, was not examined. Only participants’ declarations were used to estimate extracurricular PA and the way they spent free time. The questionnaire contained only a question about the frequency of consumption of various food products, without considering the amount. Another limitation was the anonymous nature of the survey, which prevented comparisons between dietary habits and BMI. The impact of economic standing on the formation of healthy habits is well-known, but this aspect was not taken into account in this investigation. Another limitation is that the EPA participants were also physically active during their leisure time, thus the conclusion that extended physical activity at school had an impact on BMI may be highly contested. Moreover, the investigation was undertaken during the unforeseen outbreak of the COVID-19 disease, which disrupted the research agenda. The authors are aware that lockdown restrictions limited exposure to extended PA at school and might have influenced children’s eating behaviors.

## 5. Conclusions

The study revealed that a significant proportion of 13-year-olds were overweight or obese, particularly among those who attended a standard PA. Differences in BMI and nutritional behavior were shown between adolescents with standard and extended PA at school. Extended PA, both at school and during leisure time, had a beneficial effect on BMI, but it had little effect on nutritional behavior among adolescents. The adolescents exhibited moderate nutritional behaviors; however, it was more favorable in those exposed to extended PA at school than those exposed to standard PA. Girls demonstrated more favorable behaviors than boys. Extended PA, both at school and during leisure time, was positively correlated with normal BMI and more favorable nutritional behavior, as well as negatively correlated with unfavorable nutritional behaviors. Moreover, it is possible to argue that extending organized PA at school leads to increased PA during leisure time. It is recommended to increase PA for adolescents by doubling the mandatory number of physical education lessons or other sports activities in the school curriculum.

## Figures and Tables

**Table 1:** Anthropometric indicators and BMI of the studied adolescents (the year 2021).

Measures	Groups											
	Mean Total	SPA					EPA					*p*
		Mean	Median	Min	Max	SD	Mean	Median	Min	Max	SD	
Height (cm)	167.23	167.57	166.50	147.00	186.00	8.39	166.83	167.00	146.00	182.00	7.01	0.148
Weight (kg)	59.90	61.24	58.85	37.80	109.20	14.78	58.36	57.90	29.90	93.20	120.02	0.002
BMI (kg/m^2^)	21.30	21.70	21.00	15.40	36.50	4.46	20.84	20.50	14.00	30.00	3.44	0.012
Girls												
Height (cm)	164.67	164.82	164.00	152.00	180.00	5.90	164.52	164.00	149.00	182.00	5.92	0.217
Weight (kg)	57.19	58.61	55.65	37.80	99.50	12.99	55.60	53.80	40.10	80.10	10.09	0.041
BMI (kg/m^2^) /percentile	21.06	21.58 75th	20.20	16.20	36.50	4.73	20.48 50th	20.30	14.80	29.40	3.26	0.035
Boys												
Height (cm)	169.76	170.24	170.00	147.00	186.00	9.55	169.20	170.00	146.00	182.00	7.30	0.723
Weight (kg)	62.58	63.78	61.05	37.80	109.20	16.02	61.17	61.00	29.90	93.20	13.23	0.021
BMI (kg/m^2^) /percentile	21.53	21.81 75th	21.15	15.40	33.70	4.23	21.20 50th	21.20	14.00	30.00	3.60	0.043

EPA—elevated PA group; SPA—standard PA group; *p* ≤ 0.05.

**Table 2:** BMI categories of participants by gender (the year 2021), %.

BMI Categories	Mean	Groups					
		SPA			EPA		
		Boys	Girls	*p*	Boys	Girls	*p*
Underweight	11.17	7.00	10.70	0.001	15.00	12.00	0.001
Normal	61.07	61.00	55.30	0.000	65.00	63.00	0.041
Overweight	13.63	15.00	12.50	0.024	12.00	15.00	0.034
Obesity	14.13	17.00	21.50	0.001	8.00	10.00	0.000

EPA—elevated PA group; SPA—standard PA group; *p* ≤ 0.05.

**Table 3:** Eating frequency of food products, dishes and drinks in SPA and EPA groups (the year 2021), %.

No.	Foods	Group	Frequency of Consumption [%]						*p* *
			Never	1–3 Times a Month	Once a Week	Several Times a Week	Once a Day	Several Times a Day	
1.	Milk	SPA	3.00	5.00	23.00	38.00	19.00	12.00	0.415
		EPA	2.00	6.00	15.00	36.00	29.00	13.00	
2.	Yoghurts (natural and flavoured)	SPA	13.00	18.00	17.00	41.00	7.00	5.00	0.221
		EPA	7.00	14.00	20.00	46.00	6.00	8.00	
3.	Cottage chesses	SPA	17.00	27.00	29.00	21.00	5.00	2.00	0.000
		EPA	15.00	25.00	20.00	30.00	8.00	4.00	
4.	High fat cheses (including processed and blue cheses)	SPA	4.00	11.00	23.00	43.00	16.00	4.00	0.078
		EPA	4.00	13.00	21.00	41.00	18.00	5.00	
5.	White bread and rolls	SPA	1.00	2.00	5.00	13.00	29.00	52.00	0.431
		EPA	2.00	4.00	5.00	16.00	26.00	48.00	
6.	Wholegrain bread and rolls	SPA	13.00	20.00	28.00	21.00	11.00	8.00	0.001
		EPA	15.00	23.00	15.00	26.00	13.00	10.00	
7.	White rice, white pasta, fine-grained groats	SPA	5.00	10.00	61.00	20.00	4.00	0.00	0.611
		EPA	2.00	17.00	55.00	19.00	3.00	3.00	
8.	Oatmeal, wholegrain pasta, coarse-grained groats, buckwheat groats	SPA	9.00	19.00	43.00	26.00	4.00	0.00	0.012
		EPA	1.00	17.00	57.00	17.00	4.00	5.00	
9.	Poultry dishes	SPA	3.00	10.00	43.00	36.00	7.00	2.00	0.024
		EPA	5.00	11.00	31.00	40.00	6.00	8.00	
10.	Red meat dishes	SPA	7.00	18.00	56.00	18.00	1.00	0.00	0.041
		EPA	13.00	27.00	45.00	13.00	2.00	0.00	
11.	Cold cuts, sausages, frankfurters	SPA	17.00	6.00	17.00	21.00	19.00	20.00	0.047
		EPA	11.00	6.00	16.00	33.00	19.00	16.00	
12	Canned meats	SPA	60.00	35.00	6.00	0.00	0.00	0.00	0.069
		EPA	78.00	21.00	2.00	0.00	0.00	0.00	
13.	Fried flour or meat meals	SPA	0.00	4.00	21.00	47.00	26.00	4.00	0.067
	Fried meat or flour dishes	EPA	0.00	2.00	24.00	54.00	20.00	1.00	
14.	Eggs	SPA	5.00	19.00	50.00	26.00	0.00	0.00	0.023
		EPA	7.00	18.00	59.00	10.00	6.00	0.00	
15.	Fish	SPA	20.00	30.00	49.00	1.00	0.00	0.00	0.021
		EPA	8.00	35.00	56.00	1.00	0.00	0.00	
16.	Legumes	SPA	33.00	44.00	15.00	9.00	0.00	0.00	0.067
		EPA	29.00	39.00	19.00	11.00	3.00	0.00	
17.	Fruit	SPA	3.00	3.00	8.00	29.00	26.00	33.00	0.003
		EPA	0.00	1.00	6.00	25.00	28.00	41.00	
18.	Vegetables	SPA	3.00	4.00	14.00	24.00	30.00	26.00	0.027
		EPA	2.00	1.00	17.00	10.00	25.00	36.00	
19.	Butter as an addition to bread or dishes, for frying, baking, etc.	SPA	3.00	4.00	14.00	21.00	40.00	19.00	0.044
		EPA	6.00	10.00	14.00	18.00	36.00	18.00	
20.	Lard as an addition to bread or dishes, for frying, baking, etc.	SPA	89.00	12.00	0.00	0.00	0.00	0.00	0.067
		EPA	95.00	5.00	0.00	0.00	0.00	0.00	
21.	Fast food, different types	SPA	2.00	33.00	53.00	13.00	0.00	0.00	0.041
		EPA	6.00	45.00	47.00	3.00	0.00	0.00	
22.	Sweets, candies, chocolate, bars	SPA	2.00	8.00	16.00	22.00	36.00	18.00	0.001
		EPA	2.00	13.00	21.00	36.00	17.00	12.00	
23.	Juices	SPA	10.00	19.00	45.00	21.00	3.00	4.00	0.065
		EPA	8.00	18.00	47.00	16.00	7.00	5.00	
24.	Sweetened carbonated or non-carbonated drinks	SPA	14.00	22.00	32.00	19.00	10.00	5.00	0.003
		EPA	22.00	41.00	24.00	9.00	5.00	0.00	
25.	Energy drinks	SPA	65.00	24.00	7.00	5.00	0.00	0.00	0.062
		EPA	73.00	19.00	6.00	3.00	0.00	0.00	

EPA—extended PA group; SPA—standard PA group; * chi^2^ test; *p* ≤ 0.05.

**Table 4:** Eating frequency of food products, dishes, and drinks by gender (the year 2021), %.

No.	Foods	Group	Frequency of Consumption [%]						*p* *
			Never	1–3 Times a Month	Once a Week	Several Times a Week	Once a Day	Several Times a Day	
1.	Milk	Girls	4.00	7.00	19.00	30.00	29.00	12.00	0.415
		Boys	1.00	5.00	19.00	43.00	19.00	13.00	
2.	Yoghurts (natural and flavoured)	Girls	10.00	17.00	21.00	41.00	6.00	6.00	0.061
		Boys	10.00	15.00	16.00	46.00	7.00	7.00	
3.	Cottage chesses	Girls	18.00	23.00	27.00	22.00	8.00	3.00	0.078
		Boys	14.00	29.00	22.00	29.00	5.00	3.00	
4.	High-fat chesses (including processed and blue chesses)	Girls	4.00	11.00	24.00	40.00	17.00	5.00	0.072
		Boys	4.00	13.00	19.00	44.00	17.00	4.00	
5.	White bread and rolls	Girls	1.00	4.00	4.00	20.00	31.00	42.00	0.010
		Boys	2.00	3.00	6.00	9.00	24.00	58.00	
6.	Wholegrain bread and rolls	Girls	16.00	21.00	19.00	22.00	13.00	11.00	0.023
		Boys	12.00	22.00	23.00	26.00	11.00	7.00	
7.	White rice, white pasta, fine-grained groats	Girls	6.00	15.00	56.00	18.00	6.00	1.00	0.920
		Boys	3.00	12.00	58.00	22.00	4.00	2.00	
8.	Oatmeal, wholegrain pasta, coarse-grained groats, buckwheat groats	Girls	5.00	20.00	53.00	17.00	4.00	1.00	0.028
		Boys	5.00	15.00	46.00	25.00	5.00	4.00	
9.	Poultry dishes	Girls	6.00	11.00	43.00	30.00	8.00	3.00	0.035
		Boys	2.00	10.00	39.00	37.00	5.00	7.00	
10.	Red meat dishes	Girls	9.00	26.00	54.00	12.00	0.00	0.00	0.020
		Boys	8.00	17.00	51.00	20.00	4.00	0.00	
11.	Cold cuts, sausages, frankfurters	Girls	19.00	9.00	21.00	27.00	11.00	13.00	0.001
		Boys	10.00	4.00	11.00	26.00	27.00	23.00	
12	Canned meats	Girls	70.00	29.00	2.00	0.00	0.00	0.00	0.201
		Boys	68.00	27.00	5.00	0.00	0.00	0.00	
13.	Fried flour or mest meals	Girls	0.00	6.00	24.00	48.00	21.00	3.00	0.580
		Boys	0.00	0.00	21.00	52.00	25.00	2.00	
14.	Eggs	Girls	6.00	24.00	53.00	16.00	1.00	0.00	0.068
		Boys	7.00	13.00	56.00	19.00	5.00	0.00	
15.	Fish	Girls	20.00	35.00	42.00	2.00	0.00	0.00	0.047
		Boys	13.00	35.00	52.00	0.00	0.00	0.00	
16.	Legumes	Girls	31.00	40.00	18.00	11.00	1.00	0.00	0.091
		Boys	32.00	43.00	16.00	9.00	2.00	0.00	
17.	Fruit	Girls	0.00	0.00	8.00	24.00	23.00	46.00	0.010
		Boys	3.00	4.00	6.00	30.00	30.00	28.00	
18.	Vegetables	Girls	2.00	0.00	16.00	20.00	25.00	37.00	0.023
		Boys	3.00	5.00	15.00	24.00	30.00	25.00	
19.	Butter as an addition to bread, dishes, for frying, baking, etc.	Girls	5.00	10.00	10.00	22.00	35.00	18.00	0.028
		Boys	4.00	4.00	17.00	17.00	41.00	19.00	
20.	Lard as an addition to bread, dishes, for frying, baking, etc.	Girls	90.00	10.00	0.00	0.00	0.00	0.00	0.810
		Boys	94.00	6.00	0.00	0.00	0.00	0.00	
21.	Fast food, different types	Girls	6.00	41.00	46.00	9.00	0.00	0.00	0.043
		Boys	2.00	37.00	55.00	7.00	0.00	0.00	
22.	Sweets, candies, chocolate, bars	Girls	2.00	9.00	17.00	30.00	27.00	17.00	0.240
		Boys	2.00	11.00	21.00	28.00	27.00	12.00	
23.	Juices	Girls	11.00	19.00	43.00	17.00	6.00	6.00	0.610
		Boys	7.00	18.00	49.00	21.00	3.00	3.00	
24.	Sweetened carbonated or non-carbonated drinks	Girls	23.00	33.00	29.00	10.00	5.00	2.00	0.020
	Sweetened carbonated or non-carbonated drinks	Boys	14.00	30.00	26.00	18.00	9.00	4.00	
25.	Energy drinks	Girls	76.00	19.00	4.00	2.00	0.00	0.00	0.038
		Boys	62.00	24.00	10.00	5.00	0.00	0.00	

EPA—extended PA group; SPA—standard PA group; * chi^2^ test, *p* ≤ 0.05.

## Data Availability

The data supporting reported results are available on request from corresponding author.
